# Personalized Online Learning Resource Recommendation Based on Artificial Intelligence and Educational Psychology

**DOI:** 10.3389/fpsyg.2021.767837

**Published:** 2021-12-23

**Authors:** Xin Wei, Shiyun Sun, Dan Wu, Liang Zhou

**Affiliations:** ^1^School of Communications and Information Engineering, Nanjing University of Posts and Telecommunications, Nanjing, China; ^2^Key Laboratory of Broadband Wireless Communication and Sensor Network Technology (Ministry of Education), Nanjing University of Posts and Telecommunications, Nanjing, China; ^3^Institute of Communications Engineering, Army Engineering University of People's Liberation Army of China, Nanjing, China

**Keywords:** online learning, educational psychology, artificial intelligence, student's learning ability, learning resource recommendation, LinUCB

## Abstract

The objective of the study is to explore an effective way for providing students with the appropriate learning resources in the remote education scenario. Artificial intelligence (AI) technology and educational psychology theory are applied for designing a personalized online learning resource recommendation scheme to improve students' learning outcomes. First, according to educational psychology, students' learning ability can be obtained by analyzing their learning behaviors. Their identities can be classified into three main groups. Then, features of learning resources such as difficulty degree are extracted, and a LinUCB-based learning resource recommendation algorithm is proposed. In this algorithm, a personalized exploration coefficient is carefully constructed according to student's ability and attention scores. It can adaptively adjust the ratio of exploration and exploitation during recommendation. Finally, experiments are conducted for evaluating the superior performance of the proposed scheme. The experimental results show that the proposed recommendation scheme can find appropriate learning resources which will match the student's ability and satisfy the student's personalized demands. Meanwhile, by comparing with existing state-of-the-art recommendation schemes, the proposed scheme can achieve accurate recommendations, so as to provide students with the most suitable online learning resources and reduce the risk brought by exploration. Therefore, the proposed scheme can not only control the difficulty degree of learning resources within the student's ability but also encourage their potential by providing suitable learning resources.

## 1. Introduction

Remote education is a new type of education mode in comparison with conventional face-to-face education. It enables the teachers to teach courses and the students to learn resources at remote sites. Therefore, online learning has become an important way in remote education, which especially plays an indispensable role during the 2020 COVID-19 pandemic (Wu, 2021). Compared with learning in the classroom, online learning allows students to come into contact with knowledge anytime and anywhere (George and Lal, 2019). Recently, a large number of online learning platforms that can provide students with a wide range of learning resources have emerged, such as MOOC and Coursera (Zhang et al., 2019; Jin et al., 2021). However, facing such massive learning resources, students usually cannot conveniently and effectively find the content suitable for them, resulting in inattention and low learning efficiency. Therefore, it is important to precisely locate appropriate learning resources and push them to the specific students according to their interests and personal characteristics (Wu et al., 2020b).

In fact, recommendation schemes have been used in the education field and can provide students with various forms of resources, such as articles, websites, and video courses. In Hsu et al. (2013), the authors design a reading recommendation scheme to provide articles for students that meet their reading preferences. Lichtnow et al. (2011) establishes a student-knowledge model for recommending students to some desired websites or paper links. Moreover, personalized course recommendation schemes have been concerned (Zhang et al., 2017; Pang et al., 2018; Gan et al., 2021). In general, the majority of the existing online learning resource recommendation methods are designed based on collaborative filtering algorithm. They first construct a user-item matrix and then recommend learning resources according to the similarity of users or items (Shi et al., 2014). The motivation of them is to transfer the recommended schemes in scenarios such as entertainment program watching, purchase in E-commerce into online learning. However, students are the main part of online learning. When conducting resource recommendations in online learning scenarios, two challenges are prone to happen and should be carefully concerned: (1) *How to design a recommendation scheme from the perspective of a student's own learning ability or characteristics?* (2) *How to provide students with innovative online learning resources to tap their potential?*

To deal with the first issue, several works have studied the learning behaviors of students from the perspective of educational psychology and explored the learning status of students. The goal of educational psychology is to leverage the best science in teaching-learning scenarios to better understand the psychology and practice of education. It can be used to reflect a student's psychological learning status so as to lay a foundation to explore a student's learning characteristics. For example, Bloom's model (Testa et al., 2018) can be used to explore the rules of student's acquiring knowledge. The cognitive diagnosis model derived from psychometrics (Ren et al., 2021) can classify student's mastery levels according to the composition of knowledge. The zone of proximal development (ZPD) focuses on developing a student's potential. It advises that the learning ability of students will be improved by providing more in-depth educational content (Chaiklin, 2017). Therefore, educational psychology can help us deduce hidden pedagogical laws, so as to measure student's learning status and design personalized recommendation strategies for different students.

For handling the second issue, it needs to explore new but suitable online learning resources for students from the perspective of artificial intelligence (AI). As a representative type of AI technique, recommendation approaches can effectively realize information retrieval and filtering. Specifically, in the existing recommendation scenarios, a context-based bandit algorithm, LinUCB, can achieve a balance between exploration and exploitation (Li et al., 2010). In other words, LinUCB can carefully concern users' known interests and explore users' unknown interests in the scenario of news recommendation. However, when applied to video recommendation in the online learning scenario, LinUCB still has limitations. Specifically, it does not consider student's personalized characteristics such as student's learning ability. This limitation inevitably brings a risk to the exploration process, as the recommended new resources may be much difficult for the student. It may further lead to the performance deterioration of the recommendation scheme and the increase of student's cognitive load.

To overcome the above limitations, in this article, we put forward a personalized online learning resource recommendation based on AI and educational psychology. First, from the perspective of educational psychology, we use the fraction of students participating in learning educational videos, the degree of completion, and the correct ratio of answering quizzes to estimate their ability. It can reflect their personalized learning status. Based on the ability, students can be divided into three main types by the clustering method. Second, we use the learning behavior records of all students to extract the features of the educational videos such as difficulty degrees. With the help of AI, a LinUCB-based recommendation algorithm for providing students with suitable educational videos is proposed. In this algorithm, student's ability is integrated into a personalized exploration strategy so that it can provide students with educational videos of appropriate difficulty degree. It can effectively adapt to student's learning ability and reduce the risk of exploration. It is noted that educational psychology, through which a student's distinctive ability is calculated and then integrated into a recommendation, first provides the strong possibility for a personalized recommendation. Furthermore, through the introduction of educational psychology into online learning resource recommendation, the proposed scheme in this article has higher supportability and reliability since it starts from the fundamental learning behaviors of students instead of blindly pursuing the accuracy improvement of the recommendation model. It is an effective application of research on educational psychology to online learning settings.

In summary, the contributions of this article are as follows:

1. Based on students' personal learning behavior records, we try to mine their learning ability and divide the students into groups under the guidance of educational psychology.
2. We propose a recommendation algorithm with personalized exploration under the assistance of AI. It can provide students with suitable educational videos matched with their ability, inspiring their learning potential.
3. We conduct experiments to evaluate the proposed recommendation scheme in three terms: recommendation accuracy, matching degree between student's ability and difficulty of educational video, and personalized degree of the recommendation list.

In section 2, we discuss the related works on educational psychology and recommendation systems in online learning. In section 3, we propose the personalized online learning resource recommendation scheme. In section 4, experimental results are exhibited and analyzed on the basis of educational psychology. The conclusion is provided in section 5.

## 2. Related Work

### 2.1. Educational Psychology

Educational psychology refers to the science of studying various psychological and behavioral laws in educational practice in a broad sense. It can reveal the nature of learning results, explain the process of learning, and clarify the conditions and laws of effective learning.

Zone of proximal development is an important concept in educational psychology, which refers to the gap between the two levels. One is the level of problem solving when a student conducts self-regulated learning. The other denotes the potential development level after teaching and learning. In the field of perusing cognizance, ZPD obliges that teachers ought to support students to further understand what they cannot manage without assistance (Chaiklin, 2017). Moreover, ZPD is also considered as the foundation of personalized education. In ZPD, it is imperative to provide students with learning resources that are neither too easy nor too difficult but are slightly beyond their current ability. A personalized educational scheme based on the ZPD rule is designed to maximize the cumulative gains of students (Wang et al., 2020). Meanwhile, cognitive load theory (Sweller, 1994) also indicates that the difficulty degree of learning resources should be consistent with the student's ability. Students can not remember learning resources that are too difficult in long term due to their limited working memory (Sweller, 2016). They tend to get the best learning effect when grasping learning resources slightly beyond their ability.

In addition, an important role of educational psychology is to track student's mastery of knowledge. Bloom's model describes the process of students mastering knowledge in detail with six levels, including three low levels (knowledge, comprehension, and application) and three high levels (analysis, synthesis, and evaluation). Teachers need to focus on incorporating the higher-order cognitive process into teaching and learning assessment, thereby ensuring that students are equipped with the necessary problem-solving and critical-thinking skills (Swart, 2009). The higher students have cognitive level, the higher completion the learning resources exert. Moreover, there are some other educational psychology theories to measure the learning level of students. For example, the learning curve can reflect students' learning effects. It often shows that learning counts will influence learner's mastery of knowledge. The prediction of the learning curve is the potential to present reasonable practices for personalized tutoring (Liu and Zhang, 2019). Classical test theory shows that scores can reflect the true value of students in the measured characteristics (Tatsuoka et al., 1968). In general, all the above concepts and theories provide a basis for student's learning status tracking, and ability estimation.

### 2.2. Recommendation Algorithms in Online Learning

The task of recommendations in online learning is to provide relevant learning materials to students and help them in decision making (Aguilar et al., 2017). Most existing works of recommendation algorithms in online learning can be divided into three types: user-based recommendation, item-based recommendation, and hybrid recommendation.

User-based recommendation algorithms focus on the student's characteristics. The personalized curriculum resource recommendation system in Gan et al. (2021) belongs to a learning diagnosis recommendation method combing with user interest. It can dynamically analyze user's interest preferences and find the most suitable courses for users in line with their own learning direction. Chen et al. (2019) presents an enhanced adaptive recommendation based on students' online learning style, which implements learning resource adaptation by mining students' behavioral patterns and extracting their preferences. Christudas et al. (2018) developed a way to deliver e-learning content. They integrate a compatible genetic algorithm over the learning objects and take the learning style, knowledge level, and interactivity level of the students into account. Benhamdi et al. (2017) developed a new multi-personalized recommender system for e-learning, which considers student's preferences, levels of study, and memory capacity. For user-based recommendation, most of the characteristics of students are mined according to their behavior records. Due to the uncontrollability of the behavior, only considering the characteristics of students has poor effect. It may lead to the situation that the recommended information cannot reflect their real intention.

Item-based recommendation algorithms aim at exploring the internal correlation among learning resources. Wu et al. (2020a) propose a semantic recommendation framework of learning resources based on semantic web and pedagogics. A set of reasoning rules are made from the synthesis of the type of knowledge and the internal structure of knowledge. Students are given different learning materials according to the different knowledge structures. The first literature recommendation system for datasets focuses on the connections between the literature and related datasets (Patra et al., 2020). The recommended literature may provide a detailed description and scientific finding based on that dataset, or even some prior work about the dataset's topic, which aims to increase the productivity of researchers. Kushwaha et al. (2020) use text mining tool for the enrichment of the E-textbook. They develop a phrase graph based framework to extract the mathematical concepts from the E-textbook and recommend the E-contents to the enrichment of the E-textbook. Since item-based recommendation does not need to refer to other student portraits, the system needs to have a deep understanding of the learning resource characteristics, which brings difficulties to researchers.

The hybrid recommendation algorithms combine the above two kinds of methods, considering the characteristics of students and the specific items of learning resources at the same time. Zhou et al. (2021) research the personalized learning recommendation model of student-learning resource matching, which calculates the matching degree between learners and learning resources based on the student demand model and the quality information of the learning resources. Zhang et al. (2017) propose a personalized recommendation system based on deep belief networks in the MOOC scenario. Deep belief networks can be used to extract the feature of student's attributes, behavior, and course attributes, so as to excavate the interest preference. Wan et al. (2020) construct a personalized learning resource recommendation service framework, where the data analysis layer can derive the characteristics of students, such as learning preferences and learning level. They also classify learning resources in to five categories to help students learn autonomously and efficiently. Meng et al. (2021) propose a personalized learning path generating method named learning diagnosis-learning path (LD-LP). It takes knowledge relation and student's ability into account. Obviously, the hybrid recommendation is more reliable than the other two kinds of algorithms. By considering the features of students and learning resources at the same time, it is equivalent to providing a double guarantee for the recommendation accuracy.

Compared with the user-based and item-based recommendation that only considers one side, the improved LinUCB in this article tries to take the features of students and learning resources into account at the same time, which belongs to the hybrid recommendation. As we know, LinUCB was first raised for personalized news recommendation, and it has been widely used in various research topics in recent years, such as music recommendation (Zhou et al., 2020), web services (Pilani et al., 2021), product sales (Hsu et al., 2020), and so on. However, it is rarely used in the field of education. Therefore, when it turns to the scenario of online learning, we think of designing a flexible scheme with LinUCB that can recommend learning resources suitable for students. We make full use of students' behavior information and extract the attributes of educational videos to recommend learning resources to different students according to their ability, which can control the difficulty of exploring new content to prevent them from being too far from students' ability.

## 3. Personalized Online Learning Resource Recommendation Scheme

**Figure 1:**
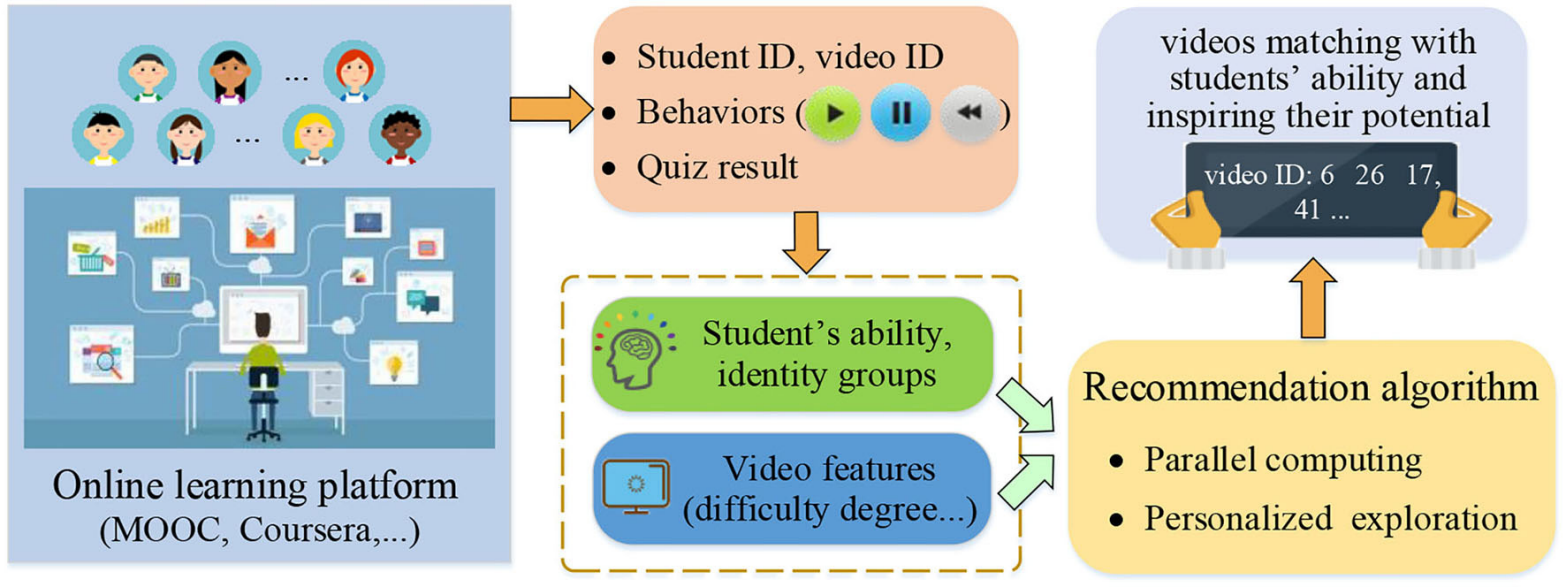
The framework of the proposed personalized online learning resource recommendation scheme.

The framework of the proposed personalized online learning resource recommendation scheme is shown in Figure 1. First, the online learning platform collects students' learning behavior logs. Then, students' learning behavior information is adopted to estimate their ability under the guidance of educational psychology. After obtaining the ability, students can be further classified into groups with different identities. Subsequently, features of educational videos, such as difficulty degree can be extracted. Finally, a LinUCB-based educational video resource recommendation algorithm is designed. Compared with existing recommendation algorithms, it uses students' ability to construct personalized exploration coefficients, providing them with suitable learning resources.

### 3.1. Dataset Collection and Preprocessing

The dataset, derived from Brinton and Chiang (2015), is collected on Coursera. Coursera is an online learning platform that provides a large number of educational video resources. There are 93 educational videos in the dataset. Each video is followed by an in-video quiz to keep abreast of the students' understanding of the video content. In other words, each student has to complete a quiz after watching each video.

Besides the quiz results, this dataset also records students' video-watching behavior logs. Specifically, it records the interaction information between students and educational videos, with a total of 29,304 student-video pairs. For each student-video pair, there are 10 pieces of learning behavior information. The fields related to the fractions for students watching the videos include: the fraction of time the student spent watching the video, the fraction of the video the student watched, and the fraction of time the student spent paused on the video. Additional fields related to interactions with the video include the number of times the student paused the video, the average playback rate that the student used while watching the video, the number of times the student skipped backward in the video, and the number of times the student skipped forward in the video. The detailed descriptions of these logs are shown in Table 1.

**Table 1:** The details of the behavior logs in the dataset.

**Field name**	**Field meaning**
userID	Student id
VidID	Educational video id
fracSpent	Fraction of the student's total time spent on watching the video (containing play, pause, rewind) to the duration of that video
fracPlayed	Fraction of the student's play and rewind time spent on watching the video (containing play, rewind) to the duration of that video
fracComp	Percentage of the video that the student plays (not containing duration of pause and rewind) to the duration of that video
numPaused	The number of times the student pauses when learning the video content
fracPaused	The fraction of the pause time to the duration of the video
numRWs	The number of times the student rewinds when learning the video content
numFFs	The number of times the student fast-forwards in the video when learning the video content
success	Whether the student has correctly answered the quiz after learning the video content (success = 1 means correct, while success = 0 means wrong)

In this article, we select several required fields from Table 1. First, compared with the quantity of *fracSpent* and *fracPlayed, fracComp* only represents the degree of completion of the video content learning excluding the repetition and pausing. Therefore, *fracComp* is more suitable to measure the time that the student spends on a video. Second, the pausing behaviors represented by *numPaused* and *fracPaused* may be caused by unexpected circumstances, such as the student leaving for something temporarily or the network is stuck. Therefore, we do not use the pausing behavior in this article. Third, in the subsequent calculation of educational video's difficulty degree, we choose *numRWs* instead of *numFFs* as the measuring factor. If rewinding behavior exists, it reflects that the video is of some difficulty, needing to be watched repeatedly to strengthen understanding. Based on the above analysis, we focus on the three main fields: *fracComp, numRWs*, and *success* in the follow-up study.

After collecting the original dataset, the next step is to perform data cleaning. Specifically, we first delete 549 log records with empty entries in all student-video pairs. Moreover, we remove 1,821 data with error time records. For example, data with too long pause time, data with too many times of fast-forwarding or rewind, *fracComp* > 1. In the end, 26,934 student-video pairs and a total of 2,219 students can be retained. With the preprocessed dataset, the important task is how to extract the characteristics of students and educational videos from the learning behavior information in student-video pairs, and recommend the most suitable educational videos for different students according to their learning characteristics.

### 3.2. Student's Ability Calculation and Identity Classification

**Figure 2:**
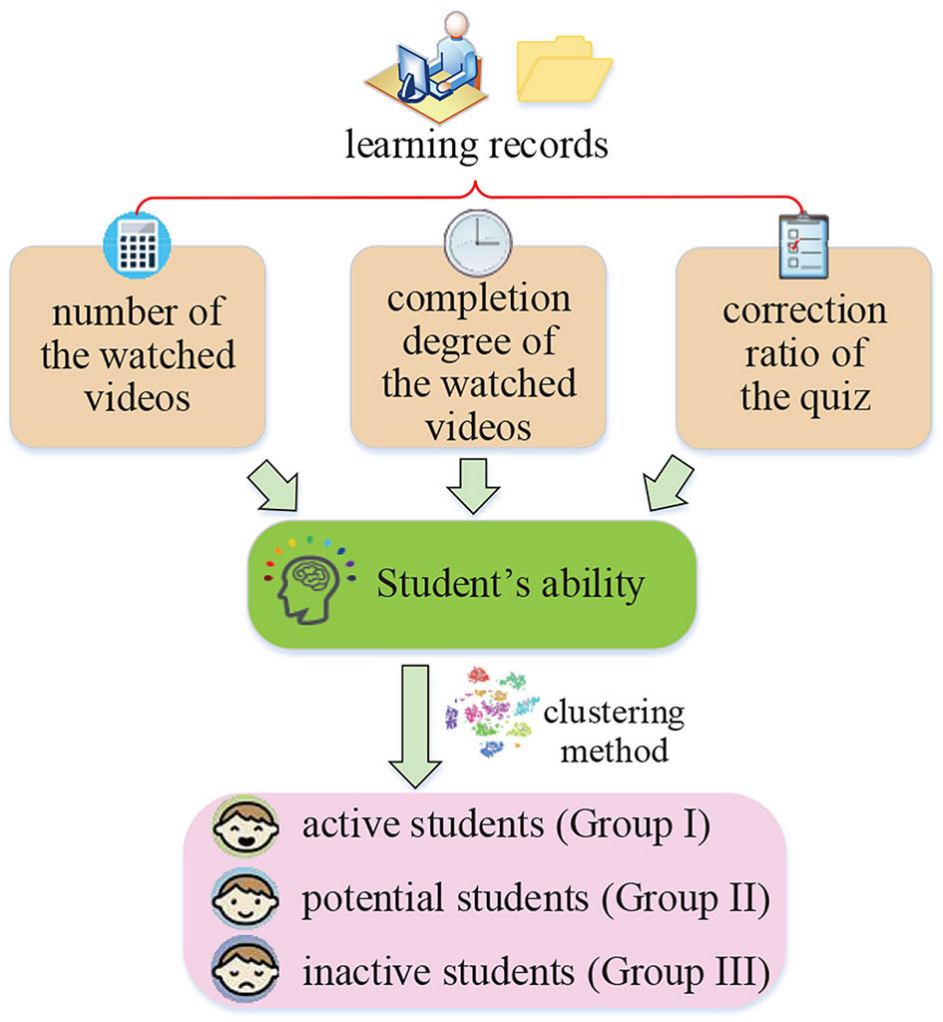
The process of student's ability calculation and identity classification.

Student's ability is an important element influencing or even determining resource recommendation effect in online learning scenarios. In order to describe student's ability, we first analyze the factors affecting ability from the perspective of educational psychology and then calculate ability from the dataset depicted in section 3.1. In addition, based on obtained ability, students can be further divided into three main groups, active students, potential students, and inactive students, which is good for the subsequent processing. The whole process of students' ability calculation and identity classification is shown in Figure 2.

#### 3.2.1. Student's Ability Calculation

The student's ability can be measured from two aspects: learning process and learning result. The learning process can be described by student's behaviors during video watching. Learning result refers to whether they finally pass the in-video quizzes. Ballera et al. (2014) mention that when it comes to answering questions related to specific knowledge, the students who answer correctly are more competent than those who answer incorrectly. The students who interact more with learning resources tend to have a better understanding of learning activities and stronger learning ability. Furthermore, educational psychology provides the theoretical basis for estimating student's ability. Bloom's model puts forward that the completion of educational content is positively correlated with student's cognitive ability (Testa et al., 2018). Classical test theory shows that scores can reflect students' mastery of knowledge and measure student's ability (Liu and Bian, 2021). Based on these theories, we calculate the student's overall ability as follows:


(1) $$\begin{array}{l} ability_{i}=k*\frac{N}{total_{num}}+l*\frac{\sum_{j=1}^{N}fracComp_{ij}}{N}+\left(1-k-l\right)\\ {}*\frac{\sum_{j=1}^{N}success_{ij}}{N}, \end{array}$$

where *ability*_*i*_ represents the overall ability of the student *S*_*i*_. *N* indicates the number of educational videos *S*_*i*_ has learned. Its ratio to the total number of educational videos *total*_*num*_ can be used to represent the participation degree of *S*_*i*_. The last two terms in Equation (1) can be used to, respectively, represent student's average completion of video content and the average correct answer rate of in-video quizzes. *k, l* are the weight values that control the importance of these three terms.

#### 3.2.2. Student's Identity Classification

In an online learning platform, the number of students is large and learning ability values among them are greatly different. If a student's identity can be known or classified, it can reflect student's learning status. The subsequent recommendation algorithm can also provide the targeted educational videos according to the identity. In this article, K-means clustering (Li et al., 2019) is used for performing a student's identity classification according to two indicators, student's ability and the number of educational videos a student has watched.

**Figure 3:**
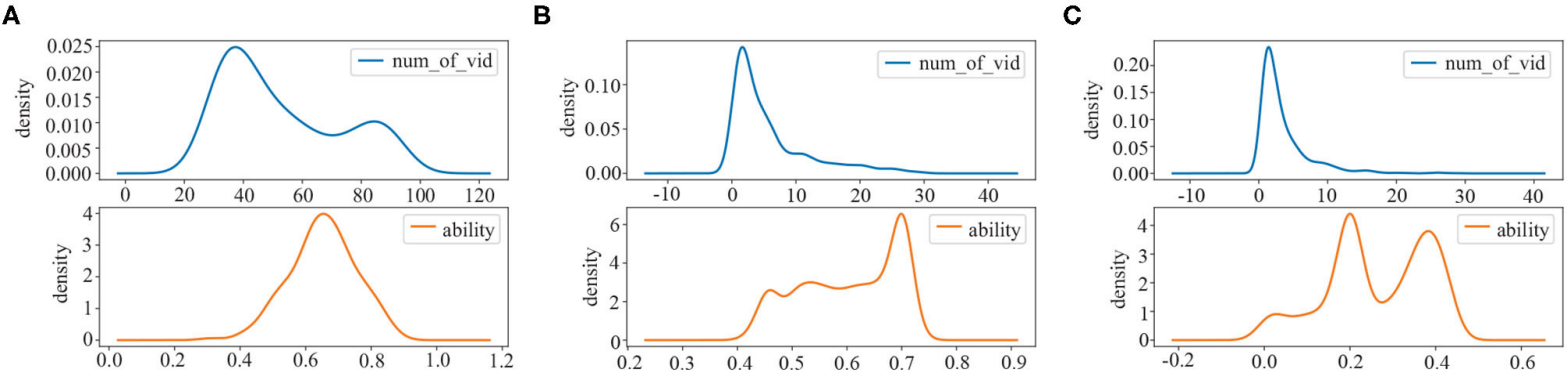
The result of student's identity clustering: **(A)** active students (Group I); **(B)** potential students (Group II); **(C)** inactive students (Group III).

In this article, we divide students into three groups. The clustering result is shown in Figure 3. The blue line represents the probability density distribution of the number of videos watched by the students in each group. The orange line indicates the probability density distribution of the student's ability in each group. First, there are 194 students in Group I. As can be seen from Figure 3A, the number of videos watched by students in this group ranges from 29 to 92 (mean = 52.88, SD = 20.002), concentrates in 30~60; The ability value ranges from 0.31 to 0.88 (mean = 0.65, SD = 0.100), concentrates in 0.53~0.76. Second, there are 1,265 students in Group II. It can be observed from Figure 3B that the number of videos studied by students in this group is between 1 and 30 (mean = 5.70, SD = 6.052), concentrates in 1~8. The ability value is between 0.40 and 0.74 (mean = 0.60, SD = 0.089), concentrates in 0.58~0.72. Third, a total of 760 students belong to Group III. From Figure 3C, the number of videos watched by this group of students ranges from 1 to 28 (mean = 3.44, SD = 3.556), while most of them are from 0 to 4. The ability value ranges from 0.01 to 0.47 (mean = 0.26, SD = 0.118), while most of them are between 0.13 and 0.44.

Through the above result and analysis, we can determine the students' identities of the three groups:

- The students in Group I tend to watch more educational videos than the other two groups and their learning ability values are always at a high level. We define students in this group as active students. In general, they not only have a high degree of participation but also have the strong ability.
- The number of educational videos watched by students in Group II is obviously decreased compared to that in Group I. However, the student's ability in Group II is still high, which is almost the same as that of Group I. We denote students in this group as potential students. Though they have only learned a small number of educational videos, their ability is high, and they have great potential to be tapped.
- The students belonging to Group III watch the least educational videos and have a relatively low ability value. Compared with the other two groups, they are not active and perform poorly throughout the learning process. We consider students in this group as inactive students. They seldom participate in learning educational videos, and their own ability is quite weak. Therefore, it is hard to dig out their learning patterns from their learning behaviors.

### 3.3. Educational Video Recommendation With Personalized Exploration

**Figure 4:**
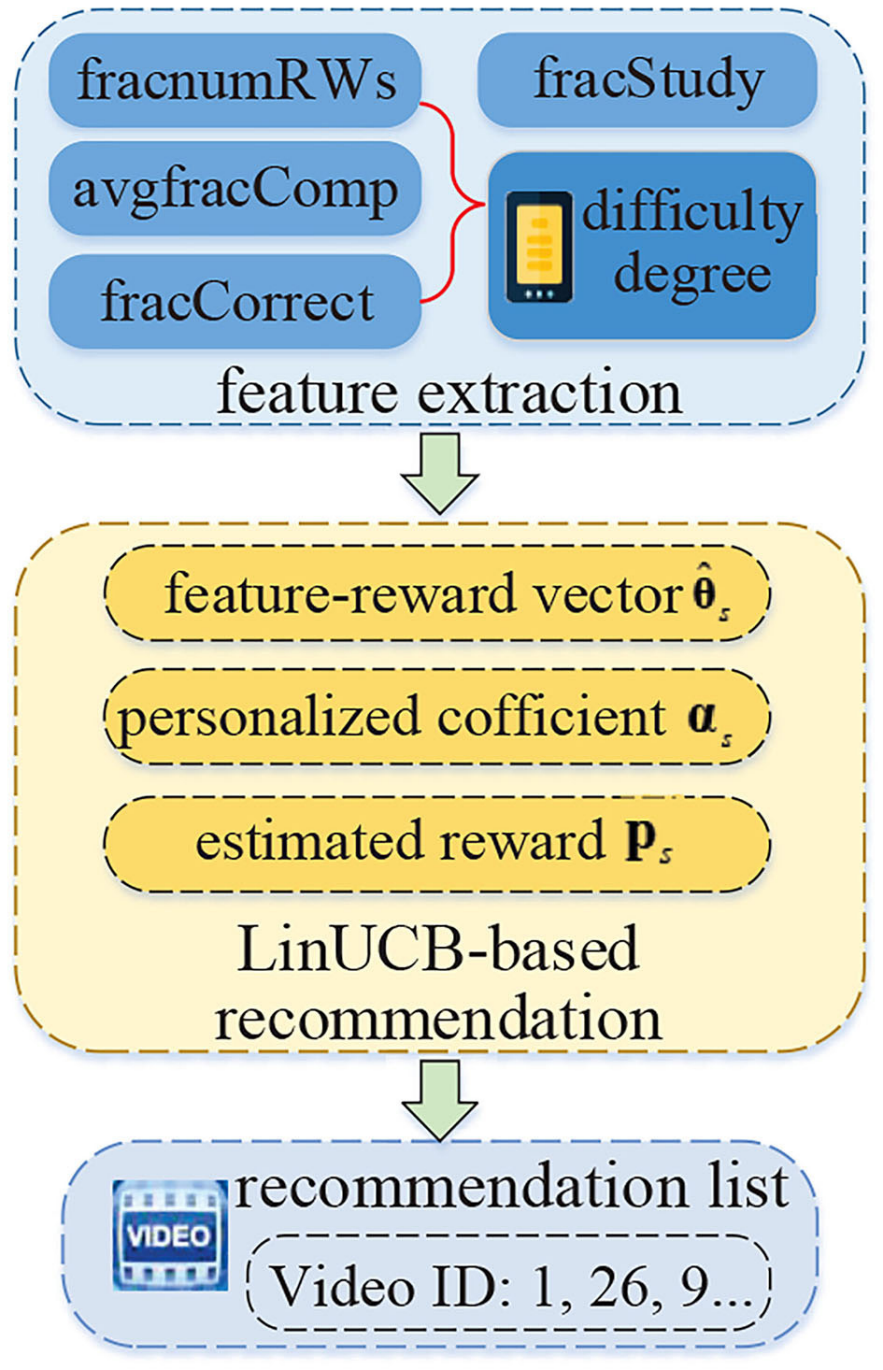
The process of educational video recommendation with personalized exploitation.

After obtaining student's ability and grouping student's identity, the key procedure is to realize online learning resource recommendations. Specifically, it can be further divided into two steps, as shown in Figure 4. Specifically, features are selected or calculated first. Then, a recommendation algorithm is proposed, which is based on the LinUCB model and considers personalized exploration according to student's ability.

#### 3.3.1. Feature Extraction

In the online learning scenarios, information that involves the student's privacy, such as gender, age, educational background, usually cannot be obtained. Considering this, feature extraction is conducted by using student's historical learning behavior records in the preprocessed dataset. In other words, based on the fields of all the students' learning behaviors, five representative and important attributes about the educational videos can be extracted, which will be taken as the input of the LinUCB-based recommendation model.

- Learning rate (*fracStudy*): It indicates the fraction of students that have learning behavior records with the specified educational video;
- Average completion degree (*avgfracComp*): It indicates the average completion ratio of all the students that have watched the video;
- Fraction of rewind times (*fracnumRWs*): It represents the fraction of the total rewind times of all students watching the video to the threshold of rewind times;
- Correct rate (*fracCorrect*): It indicates the fraction of students who have correctly answered the in-video quiz relative to the number of students watching the video.

These four attributes can be calculated as follows:


(2) $$\begin{array}{l} fracStudy_{j}=\frac{M}{total\text{\_}num\text{\_}std}, \end{array}$$

(3) $$\begin{array}{l} avgfracComp_{j}=\frac{\sum_{i=1}^{M}fracComp_{ij}}{M}, \end{array}$$

(4) $$\begin{array}{l} fracnumRWs_{j}=\frac{\sum_{i=1}^{M}numRWs_{ij}}{M*numRWs_{max}}, \end{array}$$

(5) $$\begin{array}{l} fracCorrect_{j}=\frac{\sum_{i=1}^{M}success_{ij}}{M}, \end{array}$$

where the subscript *j* represents the *j*^*th*^ educational video, and *M* represents the number of students that participate in the video *v*_*j*_. *total*_*num*_*std* is the total number of students. *fracComp*_*ij*_ represents the completion of *v*_*j*_ for *S*_*i*_. *numRWs*_*ij*_ refers to the rewind times when *S*_*i*_ watches *v*_*j*_. *numRWs*_*max*_ is the threshold of rewind times and the maximum value is 60. *success*_*ij*_ indicates whether *S*_*i*_ completes the in-video quiz correctly after learning *v*_*j*_.

Finally, the difficulty degree of each educational video is also an important attribute. In a traditional face-to-face learning scenario within the classroom, the difficulty degree of learning resources is usually determined by teachers according to their teaching experience. However, this manner is not suitable and feasible for online learning. In order to ensure the adaptability of educational video in an online learning scenario, in this article, the difficulty degree of the educational video is determined by students' learning behavior records. The calculation of this attribute is shown as:


(6) $$\begin{array}{l} dif_{j}=p*\left(1-\frac{\sum_{i=1}^{M}fracComp_{ij}}{M}\right)+q*\left(1-\frac{\sum_{i=1}^{M}success_{ij}}{M}\right)\\ \;+\left(1-p-q\right)*\frac{\sum_{i=1}^{M}numRWs_{ij}}{M*numRWs_{max}}\\ \;=p*\left(1-avgfracComp_{j}\right)+q*\left(1-fracCorrect_{j}\right)\\ \;+\left(1-p-q\right)*fracnumRWs_{j}, \end{array}$$

where *dif*_*j*_ represents the difficulty of *v*_*j*_ and the value range is 0 ~ 1. *p, q* are the weight values.

Thus, once a student generates an educational video watching record, the five attributes of the video are taken as the features of the student. Finally, each student forms a feature matrix.

#### 3.3.2. Recommendation Algorithm

In the online learning scenario, when a student makes a request to the educational website, the recommendation engine within it will retrieve that student's historical learning behavior records, analyze student's ability, and determine the appropriate educational videos. In this article, we assume that student's learning content and interest will not change in a short term, so time issue is not considered. Compared with the existing LinUCB, there have been two improvements in the proposed algorithm. First, we use a parallel matrix computation to replace the multiple serial vector computations of traditional LinUCB to improve the speed of computation and the utilization of computer resources. By using this improvement, the LinUCB can transfer the multiple serial vector computation into one parallel matrix computation for all the students. In other words, by using this improvement, all the students can get recommendation lists more efficiently when they send recommendation requests. Second, we replace the fixed parameter of traditional LinUCB with the personalized exploration coefficient, which considers the student's ability and attention scores. This consideration can help the recommendation algorithm provide educational videos with appropriate difficulty degrees according to student's ability and status. It can make the recommendation model to adapt better to exploration strategies and reduce the risk brought by exploration. The relationship between these two improvements is that they, respectively, concern recommendation issues from common and individual perspectives.

Next, we introduce the improved LinUCB-based recommendation algorithm in detail.

*Improvement I (Parallel Computing)*: To improve the computation speed and reduce the utilization cost, we combine multiple serial vector computations of the traditional LinUCB into one parallel matrix computation. In the LinUCB, it assumes that the expected value of each *r* is the linear function of its feature vector **x** for each user. In the online learning scenarios, the arms in the LinUCB refer to all educational videos provided by the online learning platform, and the user refers to the student involved in the learning of educational videos. In this article, student *s* represents the target recommendation object in the following description. Thus, the expected payoffs of all arms in the improved LinUCB are computed as follows:


(7) $$\begin{array}{l} \text{E}\left[{\text{r}}_{s}|\text{X}\right]=\text{X}{\hat{\theta }}_{s}, \end{array}$$

where ${\text{r}}_{s}={\left[{r_{s}}^{1},\ldots ,{r_{s}}^{j},\ldots ,{r_{s}}^{n}\right]}^{⊤}$. ${r_{s}}^{j}$ is the payoff about student *s* and *j*^*th*^ educational video in online learning resource set *C*. *n* denotes the number of educational videos, which can be flexibly added or reduced. $\text{X}=\left[{\text{x}}_{1}^{⊤},\ldots ,{\text{x}}_{j}^{⊤},\ldots ,{\text{x}}_{n}^{⊤}\right]$ is an *n* × *d* matrix, where ${\text{x}}_{j}^{⊤}$ is the feature vector of the *j*^*th*^ video in *C*, **x**_*j*_ = [*fracStudy*_*j*_, *avgfracComp*_*j*_, *fracnumRWs*_*j*_, *fracCorrect*_*j*_, *dif*_*j*_]. *d* refers to the number of video features, here *d* = 5. It is noted that **X** is the same for all students because it is only related to the fixed attributes of educational videos. ${\hat{\theta }}_{s}={\left[{\hat{\theta }}_{s}^{1},{\hat{\theta }}_{s}^{2},\ldots \ldots {\hat{\theta }}_{s}^{d}\right]}^{⊤}$ is a *d* × 1 coefficient vector to be learned, which aims to obtain feature-reward of each video. Its row element ${\hat{\theta }}_{s}^{k}$ refers to the parameter of the *k*^*th*^ feature. The estimated ${\hat{\theta }}_{s}$ can be calculated as:


(8) $$\begin{array}{l} {\hat{\theta }}_{s}={\left({\text{D}}_{s}^{⊤}{\text{D}}_{s}+{\text{I}}_{d}\right)}^{-1}{\text{D}}_{s}^{⊤}{\text{c}}_{s}\\ \;={\text{A}}_{s}^{-1}{\text{b}}_{s}, \end{array}$$

(9) $$\begin{array}{l} \{\begin{array}{c} {\text{A}}_{s}={\text{D}}_{s}^{⊤}{\text{D}}_{s}+{\text{I}}_{d}\\ {\text{b}}_{s}={\text{D}}_{s}^{⊤}{\text{c}}_{s} \end{array}, \end{array}$$

where ${\text{D}}_{s}=\left[{\text{x}}_{1}^{⊤},\ldots ,{\text{x}}_{i}^{⊤},\ldots ,{\text{x}}_{m_{s}}^{⊤}\right]$ is the feature matrix composed of *m*_*s*_ row vector ${\text{x}}_{i}^{⊤}$. Its dimension is *m*_*s*_ × *d*. ${\text{x}}_{i}^{⊤}$ represents the feature vector of the *i*^*th*^ video in set *M*_*s*_. *M*_*s*_ is composed of the educational videos in the learning behavior records of student *s*, and its number is *m*_*s*_. Let ${\text{c}}_{s}={\left[r_{1},\ldots ,r_{i},\ldots ,r_{m_{s}}\right]}^{⊤}$, and its row element *r*_*i*_ represents the payoff of the *i*^*th*^ video in *M*_*s*_. In particular, the payoff *r*_*i*_ is defined as the completion of educational videos for students *s*, that is, the value of *fracComp*. **A**_*s*_ is a *d* × *d* diagonal matrix. **b**_*s*_ is a *d* × 1 vector, and the row element in **b**_*s*_ denotes the cumulative payoff of each video feature.

Subsequently, according to the LinUCB, we obtain the total estimated rewards of educational videos for the student *s*:


(10) $$\begin{array}{l} {\text{p}}_{s}=\text{X}{\hat{\theta }}_{s}+\alpha_{s}⊙\sqrt{{\left(\text{X}{{\text{A}}_{s}}^{-1}{\text{X}}^{⊤}\right)}_{diag}}, \end{array}$$

where **p**_*s*_ is an *n* × 1 vector whose row elements represent the total estimated rewards of each video in *C*. ***α***_*s*_ is also an *n* × 1 vector that can control whether the decision is inclined to exploitation or exploration. The smaller ***α***_*s*_ is, the larger the probability of selecting recommended videos from the watched videos becomes. On the contrary, the larger ***α***_*s*_ is the more likely to recommend new videos that student *s* has not watched. ${\left(\text{X}{{\text{A}}_{s}}^{-1}{\text{X}}^{⊤}\right)}_{diag}$ is an *n* × 1 vector whose row elements are diagonal elements of $\text{X}{{\text{A}}_{s}}^{-1}{\text{X}}^{⊤}$. This vector indicates the predicted variance of the expected payoffs $\text{X}{\hat{\theta }}_{s}$. The Hadamard product of ***α***_*s*_ and $\sqrt{{\left(\text{X}{{\text{A}}_{s}}^{-1}{\text{X}}^{⊤}\right)}_{diag}}$ can be explained as the uncertainty of $\text{X}{\hat{\theta }}_{s}$. Therefore, in Equation (10), the former item represents the exploitation of what students have watched, and the latter item realizes the guaranteed exploration of new educational videos.

*Improvement II (Personalized Exploration Strategy)*: In order to adapt to the ability of different students and perceive their acceptance of educational videos with different difficulty degrees, we use the attention mechanism to calculate the parameter ***α***_*s*_ that control the ratio of exploration and exploitation. Specifically, ***α***_*s*_ denoted as personalized exploration coefficient is related to the student's ability and attention and can reflect the personalized needs of different students. It is defined as:


(11) $$\begin{array}{l} \alpha_{s}=\frac{ability_{s}}{n}*{\text{S}}_{s}, \end{array}$$

where *ability*_*s*_ represents the ability of the student *s*, which can be estimated by Equation (1). It can reflect the student's acceptance of videos with different difficulty degrees and determine whether the student can actually master the content in the videos with unknown difficulty degrees. The student's need for exploration increases with the ability. **S**_*s*_ is an *n* × 1 vector that refers to student's attention degrees for each video and a reflection of the student's thirst for unknown knowledge. By using the attention mechanism, **S**_*s*_ can be calculated as follows:


(12) $$\begin{array}{l} {\text{S}}_{s}=D_{n\times m_{s}}*{\text{c}}_{s}, \end{array}$$

where *D*_*n*×*m*_*s*__ is an *n*×*m*_*s*_ dissimilarity matrix and each row vector of *D*_*n*×*m*_*s*__ refers to the vector of dissimilarity values between each video in *C* and *M*_*s*_. The element in the *l*^*th*^ row and *m*^*th*^ column of *D*_*n*×*m*_*s*__ represents the Euclidean distance between the difficulty of the *l*^*th*^ video in *C* and the *m*^*th*^ video in *M*_*s*_. The calculation of *D*_*n*×*m*_*s*__ is as follows:


(13) $$\begin{array}{l} D_{n\times m_{s}}\left[l,m\right]=\sqrt{{\left(dif_{l\in C}-dif_{m\in M_{s}}\right)}^{2}}, \end{array}$$

where *D*_*n*×*m*_*s*__ sets the difficulty dissimilarity value between the videos that students have watched and the videos in *C*. **c**_*s*_ performs a weighted summation of the row elements in *D*_*n*×*m*_*s*__ to obtain the student's attention degree for each educational video. Therefore, **S**_*s*_ reduces the attention degrees of the videos that the student *s* has watched and increases the attention weights of the videos that have not been watched. The greater the dissimilarity in difficulty degree between the videos have and have not been watched, the easier it is to attract student's attention, and the higher the desire of the student to explore the unknown knowledge.

For each student, *ability*_*s*_ and **S**_*s*_ are different. Therefore, for one thing, the personalized coefficient we designed can make full use of a student's learning state to adjust the ratio of exploration adaptively according to the student's ability and attention. For another, the personalized ***α***_*s*_ can ensure that the difficulty degree of the explored educational videos is within the range of the student's ability so as to reduce the risk of exploration.

Finally, we choose top-N videos with the highest values in *p*_*s*_ to form a recommendation list,


(14) $$\begin{array}{l} \text{list}\left[v\right]=\text{to}{\text{p}}_{N}\left(p_{s,v}\right),{\text{p}}_{s}=\left[p_{s,v}\right], \end{array}$$

where *p*_*s,a*_ denotes the elements in vector **p**_*s*_. Algorithm 1 outlines the whole recommendation algorithm.

**Algorithm 1:** Educational video recommendation algorithm with personalized exploration.

**Input:** *X, D*__*s*__, *c*_*s*_, *ability*_*s*_, *dif*_*l* ∈ *C*_, *dif*_*m* ∈ _*M*__*s*__ **Output:** *p*_*s*_, list[*v*]
1: **for** each student *s* **do**
2: **Calculate the feature-reward vector:**
3: ${\text{A}}_{s}={\text{D}}_{s}^{⊤}{\text{D}}_{s}+{\text{I}}_{d}$
4: ${\text{b}}_{s}\;={\text{D}}_{s}^{⊤}{\text{c}}_{s}$
5: ${\hat{\theta }}_{s}={\text{A}}_{s}^{-1}{\text{b}}_{s}$
6: **Calculate the personalized exploration coefficient:**
7: $D_{n\times m_{s}}\left[l,m\right]=\sqrt{{\left(dif_{l\in C}-dif_{m\in M_{s}}\right)}^{2}}$
8: **S**_*s*_ = *D*_*n*×*m*_s__*c_*s*_
9: $\alpha_{s}=\frac{ability_{s}}{n}*{\text{S}}_{s}$
10: **Obtain the total estimated rewards of all educational videos:**
11: ${\text{p}}_{s}=\text{X}{\hat{\theta }}_{s}+\alpha_{s}⊙\sqrt{{\left(\text{X}{{\text{A}}_{s}}^{-1}{\text{X}}^{⊤}\right)}_{diag}}$
12: **Obtain the personalized recommendation list:**
13: list[*v*] = top_*N*_(*p*_*s, v*_), ***p***_*s*_ = [*p*_*s,v*_]
14: **end for**

## 4. Experiment and Discussion

### 4.1. Evaluation Index

The evaluation index selected in this article can be divided into two categories. Precision, recall, F1, and hit_ratio are commonly used as accuracy-related evaluation indicators in recommendation systems. They aim to measure whether the recommended videos correctly hit the learning characteristics of students. Adaptivity and personalization focus on measuring the characteristics of the recommendation results. Furthermore, adaptivity belongs to the individual level non-accuracy-related index since it can reflect whether the recommendation results are well-adapted to the individual student. Personalization belongs to the system level non-accuracy-related index because it represents the overall diversity of the recommendation algorithm. The specific meanings of these evaluation indexes are as follows.

- ***precision@N:*** It refers to the ratio of successfully recommended educational videos among *R*(*s*). In general, it takes the recommendation list as the reference standard, which refers to the proportion of educational videos recommended to students that belong to their actual video-watching set. The successful recommendation here not only means that the student watches the video but also correctly completes the in-video quiz corresponding to the video.
  (15) $$\begin{array}{l} \text{precision@N}=\frac{\sum_{s}|T\left(s\right)\cap R\left(s\right)|}{\sum_{s}|R\left(s\right)|}, \end{array}$$
  where *T*(*s*) denotes the set of educational videos watched by the student *s* and the corresponding in-video quiz correctly answered. *N* = |*R*(*s*)|, is a constant and represents the length of the recommendation list.
- ***recall@N:*** It is based on the students' actual video-watching set, which refers to the ratio of successfully recommended educational videos among *T*(*s*).
  (16) $$\begin{array}{l} \text{recall@N}=\frac{\sum_{s}|T\left(s\right)\cap R\left(s\right)|}{\sum_{s}|T\left(s\right)|}. \end{array}$$
- ***F1@N***: It comprehensively evaluates the precision and recall, which is the harmonic mean of *precision@N* and *recall@N*.
  (17) $$\begin{array}{l} \text{F1@N}=\frac{2*\text{precision@N}*\text{recall@N}}{\text{precision@N}+\text{recall@N}}. \end{array}$$
- ***hit_ratio@N***: It refers to the ratio of success times in the total times of recommendations. For the student *s*, when *R*(*s*) overlaps with *T*(*s*), this recommendation is considered a success.
- ***adaptivity@N***: It refers to the difference between the average difficulty degree of the educational videos in the recommendation list *D*(*R*_*s*_) and that of the videos the student has watched *D*(*N*_*s*_). This evaluation index is used to measure whether the recommended educational videos are in line with the level of student's the ability, reflecting the adaptivity of the recommendation results for an individual student. According to the fit between the recommended content and student's learning ability, adaptivity may also determine whether students are willing to follow the recommendation system. Good adaptivity is conducive to strengthening student's trust in the recommendation system.
  (18) $$\begin{array}{l} \text{adaptivity@N}=\frac{\sum_{s}^{\left|S\right|}\left[D\left(R_{s}\right)-D\left(N_{s}\right)\right]}{\left|S\right|}, \end{array}$$
- ***personalization@N***: It describes the average differences among the obtained recommendation lists of different students. It represents the overall diversity of the recommendation scheme. The higher this value is, the more personalized characteristic the recommendation scheme has. A high personalization means that the recommendation can provide a personalized experience for each unique student.
  (19) $$\begin{array}{l} \text{personalization@N}=\frac{2}{|S|\left(|S|-1\right)}\underset{s_{1}\neq s_{2}}{\sum }\left(1-\frac{\left|R\left(s_{1}\right)\cap R\left(s_{2}\right)\right|}{N}\right), \end{array}$$
  where *s*_1_, *s*_2_ are two different students, *s*_1_, *s*_2_ ∈ *S*. *R*(*s*_1_) and *R*(*s*_2_) are the recommendation lists provided by Algorithm 1 for student *s*_1_ and student *s*_2_, respectively.

### 4.2. Performance Analysis

The dataset used in the experiment has been introduced in section 3.1. In this article, we respectively, analyze the evaluation index for the active students, potential students, and inactive students classified in section 3.2.2. In particular, we delete 2,582 logs with almost zero participation from the 26,934 available learning behavior logs. Finally, the number of active students, potential students, and inactive students in this experiment are 194, 890, and 517, respectively. The corresponding number of logs about these three groups of students are 10,258, 10,932, and 3,162.

#### 4.2.1. Performance Analysis of Basic Functions

**Figure 5:**
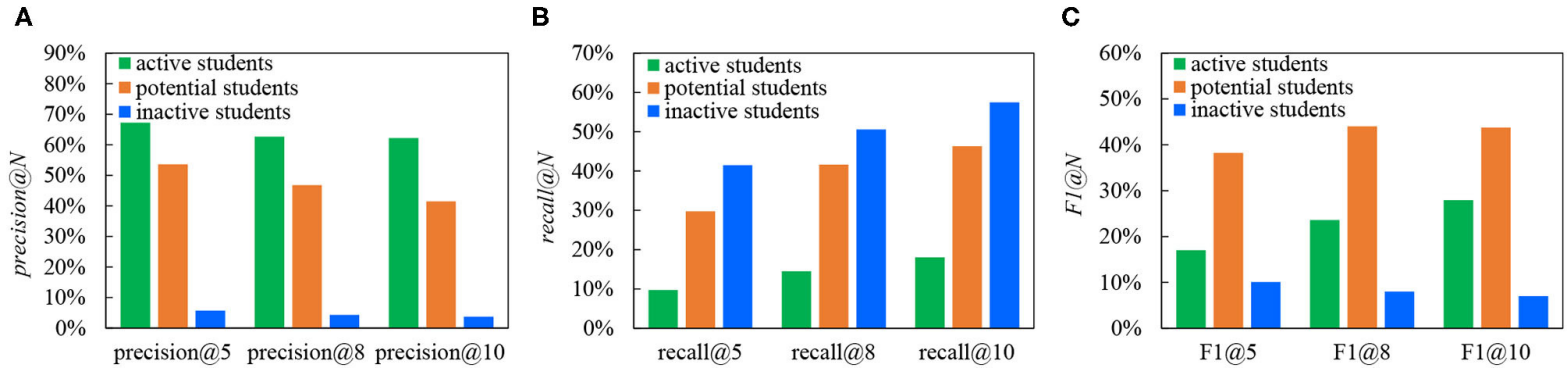
*Precision@N, recall@N*, and *F1@N* of three groups by the proposed recommendation algorithm, **(A)** *precision@N*, **(B)** *recall@N*, **(C)** *F1@N*.

Figure 5 exhibits the performance of active students, potential students, and inactive students in *precision@N, recall@N*, and *F1@N*. There are two main goals of the experiment in this article. The one is to measure the accuracy of our recommendation scheme. The other is to compare the differences of these indicators in different student groups and explore the reasons for these differences. As can be seen from Figure 5A, *precision@N* decreases with the increase of *N*. The *precision@N* of active students is always the highest, followed by potential students and inactive students. Since active students provide relatively fruitful learning behaviors, the proposed recommendation algorithm in section 3.3.2 can describe their learning status more precisely. In addition, from Figure 5B, the *recall@N* of the active students is generally low, while that of inactive students is high. This is consistent with the characteristics of the three student groups analyzed in section 3.3.2. Active students have watched the majority of videos, while potential students and inactive students have watched a much smaller number of videos than active students. Especially for inactive students, most of them only learn 0~4 videos. As a result, when calculating *recall@N*, if *T*(*s*) in the denominator of Equation (16) is small, the value of *recall@N* is large. Although for active students, the number of successfully recommended videos is more than that of the other two kinds of students, this value is still smaller compared with the dozens of videos they have watched. That is the reason for low *recall@N* for active students.

The results in Figure 5C show that the potential students have the highest *F1@N*. We mainly make a horizontal comparison of *F1@N* in combination with the characteristics of three groups. (1) The *F1@N* of active students keeps increasing. The larger the value of *N* becomes, the better performance can be obtained, as more videos have been watched by the students. (2) Both *F1@8* and *F1@10* of potential students are relatively high since the majority of potential students have watched 1~10 educational videos. As the number of recommended videos is too much for them (e.g., N ≥ 10), *F1@10* has begun to decline slightly. (3) *F1@N* for inactive students is always falling because they watch a small number of videos (0 ~ 4) and have the relatively weak ability. Based on the above analysis, when the length of the recommendation list is set, *precision@N* can evaluate the accuracy of the recommendation algorithm more objectively and reasonably. It refers to the ratio of successfully recommended educational videos in the recommendation list, which is not affected by the student's identity.

Table 2 exhibits the *hit_ratio@N* of three different groups, which is used to reflect the probability of a successful experiment. The horizontal comparison reflects that the hit_ratio increases with the increase of *N* since it is a cumulative value. Otherwise, it can be observed that the *hit_ratio@N* of both active students and potential students are very high, where potential students are 0.84, 2.66, and 1.23% higher than active students in *hit ratio@5, hit ratio@8*, and *hit ratio@10*, respectively. The reason is that they provide enough behavior logs for us to mine their learning rules so as to greatly improve the possibility of successful recommendations. On contrary, the *hit_ratio@N* of inactive students is much lower than those of the other two groups due to their low participation and weak ability. It is difficult to sum up learning patterns from them and precisely recommend educational videos for them.

**Table 2:** *Hit_ratio@N* of three groups by the proposed recommendation algorithm.

**Student group**	**hit_ratio@N**		
	**hit_ratio@5 (%)**	**hit_ratio@8 (%)**	**hit_ratio@10 (%)**
Active students	94.33	95.88	97.42
Potential students	95.17	98.54	98.65
Inactive students	28.63	32.30	35.98

In a word, the results of accuracy-related indexes are all telling a fact that active students who have high participation and strong ability perform better in the learning process and can achieve more accurate recommendations. This proves that a student's learning status evaluated by educational psychology plays a part in the recommendation algorithm. Besides, the cluster results originated from educational psychology can help analyze the different recommendation performances among different student groups.

#### 4.2.2. Performance Analysis of Personalized Exploration Improvement

Next, we evaluate the performance of the improved personalized exploration strategy in these three groups. The results of *adaptivity@N* are shown in Table 3. According to ZPD theory (Chaiklin, 2017), the content to be learned in the next stage needs to have some challenges for the students, which can inspire their enthusiasm, encourage their potential, and achieve a higher level of cognition. The experiment part of adaptivity aims to check whether the recommended content matches the students' ability and complies with ZPD theory. Combined with the view of ZPD, we specifically analyze the adaptivity of the proposed personalized exploration improvement to three types of student groups:

1. For the active students, the average difficulty degree of the educational videos recommended to them is about 0.08 ~ 0.14 higher than the average difficulty of the videos the students have watched. This result is reasonable for active students with strong ability. In other words, the proposed personalized exploration improvement provides content with a bit more difficulty degree to encourage the potential of these students, exploring the highest points of their ZPD.
2. For the potential students, due to their strong ability, the difficulty degree of the recommended videos is also slightly higher than that of videos they have watched. However, the gap is smaller than that of active students, in the range of 0.06 ~ 0.09. The reason is that the potential students watch less educational videos so that slight difficulty enhancement can effectively inspire their enthusiasm and encourage them to participate more in learning. In general, it is beneficial to motivate their potential by slowly increasing the difficulty degree of online learning resources.
3. For the inactive students, some recommended educational videos have slightly higher degrees of difficulty, while the others still have difficulty degrees within the scope of their ability. Overall, the value ranges from -0.01 to 0.02, which is consistent with their own weak ability. In other words, the difficulty degree of the online learning resources should not be too far away from their ability, otherwise, it may cause their cognitive load.

**Table 3:** *Adaptivity@N* of three groups after performing personalized exploration.

**Student group**	**adaptive@5**	**adaptive@8**	**adaptive@10**
Active students	0.1396	0.1109	0.0898
Potential students	0.0883	0.0893	0.0617
Inactive students	−0.0140	−0.0130	0.0136

Therefore, the personalized exploration improvement makes our proposed scheme equipped with good adaptivity. The results for different types of student groups are highly consistent with ZPD theory in educational psychology, which aims to motivate student's potential but not increase their cognitive burden.

Finally, in order to verify whether the designed personalization coefficient ***α***_*s*_ can improve the personalization degree of recommendation algorithm, we select the active students with the highest *precision@N* from the three groups and further analyze the influence of different ***α***_*s*_ on *personalized@N*. The experiment for personalization evaluation is designed to validate that our recommendation scheme can make targeted recommendations for students with different abilities. In this article, we set three different exploration coefficients. The first one has no specific value, α = 1, in which case the degree of exploration and exploitation is the same without adjustment. The second one is a fixed value, α = 0.5, which means the ratio of exploration to exploitation is 0.5 for each student, regardless of the characteristics of the students. The third one is ***α***_*s*_ proposed in section 3.3.2. The result is shown in Table 4. It can be observed that, compared with α = 1, when α = 0.5, *personalization@5, personalization@8*, and *personalization@10* have improved by 6.55, 3.05, and 2.22%, respectively. This proves that the exploration coefficient can improve the *personalization@N* of recommendation results. Furthermore, compared with the fixed exploration coefficient of 0.5, the personalized parameter proposed in section 3.3.2 has 10.53, 13.92, and 13.51% improvements in *personalization@5, personalization@8*, and *personalization@10*, respectively. This proves that integrating student's ability into the exploration coefficient can enhance the personalization ability of the recommendation algorithm. In the same sense, the student's ability calculated according to educational psychology plays a big role in the personalized exploration strategy.

**Table 4:** *Personalization@N* of the proposed recommendation algorithm under different exploration coefficients.

**Exploration coefficient**	**personalization@5 (%)**	**personalization@8 (%)**	**personalization@10 (%)**
No specific value(α = 1)	38.15	36.78	36.49
Fixed value(α = 0.5)	44.70	39.83	38.71
**Personalized parameter**	**55.23**	**53.75**	**52.22**

*The bold values mean the Personalization@N of the recommendation algorithm under the proposed exploration coefficient*.

### 4.3. Performance Comparison With the Other Recommendation Schemes

In order to verify the effectiveness of the proposed recommendation scheme, we still choose the active students as the target to compare with some competing recommendation schemes. The schemes are as follows:

**Popular:** It is the most common recommendation scheme, which is to recommend some of the most-watched educational videos to students. This is similar to traditional offline teaching to a certain extent, in which teachers count the learning status of all students and teach the most demanding courses according to the needs of most students.

**User-based CF:** It provides recommendations according to the videos watched by some students similar to the target student. The similarity is calculated by analyzing students' learning behavior log records.

**Item-based CF:** Its principle is that the target student may prefer some videos similar to ones that the target student has watched. The similarity between videos should be calculated by analyzing the student's learning behavior log records.

We select *precision@N, recall@N, F1@N*, and *hit_ratio@N* as evaluation indexes. As shown in Table 5, our scheme has shown better performance in many ways. For the popular recommendation scheme, it does not consider any characteristics of students, thus, it has the poorest performance. Especially in precision, the proposed scheme has great improvements of 43.3 and 42.32% in *precision@8* and *precision@10*, respectively. For the item-based CF, the result is also relatively poor to the proposed scheme. For example, the proposed scheme has the smallest performance improvement of 1.88% in *recall@10* and the largest improvement of 37.1% in *precision@10*. This proves that similar items may not arouse students' interests, but new or challenging ones have a positive effect on the recommendation accuracy. Among the three competing schemes, the user-based CF has the best performance. Compared with it, the proposed scheme has 16.06, 1.08, 0.23, and 24.89% improvements in *precision@8, recall@8, F1@8*, and *hit_ratio@8*, respectively. There are also 19.22, 0.48, 2.75, and 16.37% improvements in *precision@10, recall@10, F1@10*, and *hit_ratio@10*, respectively. This just proves that user similarity cannot achieve accurate recommendations, and it is also necessary to deeply explore students' learning characteristics as the most important factor. The three comparison recommendation schemes don't consider the personalized characteristics of students, which results in the underutilization of students' information. On contrary, our proposed scheme focuses on digging the learning status of students, which emphasizes the advantages of introducing educational psychology. In a word, the comparison results and analysis show that the proposed recommendation scheme in this article can provide students with the most suitable online learning resources, and the risk brought by exploration can also be reduced.

**Table 5:** *Precision@N, recall@N, F1@N, hit_ratio@N* of different recommendation schemes.

**Evaluation index**	**Popular (%)**	**User-based CF (%)**	**Item-based CF (%)**	**Proposed (%)**
precision@8	19.39	46.63	29.95	**62.69**
precision@10	18.92	42.02	24.14	**61.24**
recall@8	14.89	15.58	13.76	**16.66**
recall@10	18.20	17.55	16.15	**18.03**
F1@8	19.20	23.36	18.87	**23.59**
F1@10	18.55	24.76	19.35	**27.51**
hit_ratio@8	81.96	70.99	63.35	**95.88**
hit_ratio@10	84.02	81.05	70.02	**97.42**

*The bold values mean the Precision@N, recall@N, F1@N, hit_ratio@N of the proposed recommendation scheme*.

### 4.4. Discussion

To sum up, through the above experimental evaluation, the results show that the proposed personalized online learning resource recommendation scheme has high precision, adaptivity, and personalization. Specifically, the experimental results on basic functions show that our recommendation scheme can accurately find suitable learning resources, in which the highest precision has arrived at nearly 70% (for active students). Combined with the educational psychology and characteristics analysis of three groups, we find active students with higher participation and stronger ability tend to get more precise recommendation results than potential students and inactive students. The experimental results on personalized exploration improvement show that the difficulty degree of learning resources provided by the proposed scheme have been controlled within the scope of student's ability, which validates its adaptivity and complies with ZPD theory in educational psychology. Meanwhile, the personalization ability of our scheme is also improved by the personalized exploration strategy, which has enhanced more than 10% compared with the originally fixed coefficient. By comparing with existing state-of-the-art recommendation schemes, our proposed scheme performs better and can achieve more accurate recommendations. Therefore, the proposed scheme in this article can provide students with suitable learning resources to motivate their potential and reduce their cognitive load under the guidance of educational psychology and AI.

It is particularly worth mentioning that educational psychology has made a significant contribution to the superior performance of our proposed scheme. Tracing back to educational psychology, it is important that the proposed scheme not only pays much attention to student's learning status but also considers the features of online learning resources. Specifically, from the student's perspective, we use information about learning procedures to analyze students' characteristics and estimate their abilities. As we know, Bloom's model claims that if students have high completion about the learning resources, they will also obtain high cognitive level (Testa et al., 2018). Classical test theory shows that scores can reflect a student's mastery of knowledge to a certain extent and can be used to estimate a student's ability (Tatsuoka et al., 1968). Therefore, the scheme we proposed can track student's learning status and use their personal information to make a timely cognitive diagnosis. More importantly, learning resources should be tailored to the student's ability. They can have a certain degree challenging to make the student's cognitive level high but not detach from their ability. According to the adaptivity analysis in section 4.2.2, for all the groups, the proposed scheme is highly adaptable. It can provide students with resources meeting their learning needs. At the same time, the personalization of the recommendation algorithm also reflects the attention to the characteristics of students. Students with different abilities can get different recommendation lists. From the perspective of learning resources, we extract various features of the educational videos for performing recommendations. It is noted that the difficulty degree of the learning resources is derived from learning behavior records of all the students, not obtained by teacher's experience. This is a more reliable and objective way. The derived difficulty degree conversely represents the overall learning status of students for the resources. According to the cognitive load theory, the difficulty degree of learning resources should be consistent with student's ability (Christudas et al., 2018). Consistent with this theory, the educational videos provided by the proposed recommendation algorithm can match well with the student's ability, not causing their cognitive load during online learning.

## 5. Conclusion

In this article, we put forward a personalized online learning resource recommendation based on AI and educational psychology. The key insight is that the difficulty degree of recommended learning resources should be matched with the student's ability according to theories in educational psychology. Under this guidance, students' ability is found and their identity can be divided according to their learning behavior records. Then, the recommendation algorithm with personalized exploration improvement has been designed by considering both student's ability and the features of online learning resources. Experimental results show that the proposed scheme is of good precision, adaptivity, and personalization. It can provide the students with suitable educational videos to motivate their potential and reduce their cognitive load.

Also, the present article leaves several open and interesting questions. First, we assume that the student's learning ability and interest do not change in a short term. As we know, these factors are varying over a long time, which should be deeply considered. Second, besides students' ability and learning status, their learning experience is also an important indicator influencing the final outcome. Therefore, online learning resource recommendations based on students' learning experience should also be paid much attention. Third, we try to solve the precise online learning recommendation issue here with the help of educational psychology theory and AI techniques. There still exist other problems that can be handled in this way, such as student's learning effect evaluation, and interactive teaching method design. We will carefully study them in our ongoing works.

## Data Availability Statement

Publicly available datasets were analyzed in this study. This data can be found here: [Github] https://github.com/emadbutt17/Student-Video-Behavior-Performance.

## Author Contributions

XW was in charge of the study design. XW and SS were responsible for the literature review. SS and DW were in charge of the coding and assessment. The first draft of the manuscript was written by XW and SS, and subsequently extended and refined by LZ. All authors read and approved the final version of the manuscript.

## Funding

This work is partly supported by the National Natural Science Foundation of China (Grant No. 62071254), the Education Reform Foundation of Jiangsu Province (Grant No. 2021JSJG364), the Teaching Reform Research Project of Nanjing University of Posts and Telecommunications (Grant Nos. JG00215JX01, JG00220JX02), and the Priority Academic Program Development of Jiangsu Higher Education Institutions.

## Conflict of Interest

The authors declare that the research was conducted in the absence of any commercial or financial relationships that could be construed as a potential conflict of interest.

## Publisher's Note

All claims expressed in this article are solely those of the authors and do not necessarily represent those of their affiliated organizations, or those of the publisher, the editors and the reviewers. Any product that may be evaluated in this article, or claim that may be made by its manufacturer, is not guaranteed or endorsed by the publisher.
